# Pristinamycin-antibiotic combinations against methicillin-resistant *Staphylococcus aureus* recovered from skin infections

**DOI:** 10.1186/s12879-025-11433-0

**Published:** 2025-09-16

**Authors:** Muath Suliman, Amr S. Bishr, Sally T. K. Tohamy, Samar S. Mabrouk, Nasser S. M. Ismail, Abdallah M. Samy, Khaled M. Aboshanab

**Affiliations:** 1 Department of Clinical Laboratory Sciences, College of Applied Medical Sciences, King Khalid University, P.O. Box 61413, 9088 Abha, Saudi Arabia; 2 Department of Microbiology and Immunology, Faculty of Pharmacy, Ain Shams University, Cairo, 11566 Egypt; 3 Department of Microbiology and Immunology, Faculty of Pharmacy (Girls), Al-Azhar University, Cairo, 11651 Egypt; 4 Department of Microbiology, Faculty of Pharmacy, Ahram Canadian University (ACU), 6Th October City, Giza, 12566 Egypt; 5 Department of Pharmaceutical Chemistry, Faculty of Pharmacy, Ain Shams University, Cairo, 11577 Egypt; 6 Medical Ain Shams Research Institute (MASRI), Faculty of Medicine, Ain Shams University, Cairo, 11577 Egypt; 7 Faculty of Science, Ain Shams University, Abbassia, Cairo, 11566 Egypt

**Keywords:** Pristinamycin, MRSA, Macrolide-resistance, Antibiotic combination, Skin infection

## Abstract

**Background:**

Macrolide-resistant and methicillin-resistant *Staphylococcus aureus*, particularly those exhibiting pristinamycin resistance, impose significant medical health consequences with limited therapeutic options. This study is designed to determine their prevalence in a major tertiary care hospital in Egypt, antimicrobial susceptibility and evaluate various pristinamycin (PST)-antibiotic combinations.

**Methods:**

Standard procedures were employed for isolation, identification, antimicrobial susceptibility, and molecular analysis of key macrolide- and methicillin-resistant genes. Phenotypic relatedness and antibiotic combinations of pristinamycin with other antimicrobial agents were done using the heatmap analysis and checkerboard assay.

**Results:**

Out of 154 positive cultures of *S. aureus* were collected from different types of skin infections. The lowest resistance was shown for linezolid (5.2%), followed by vancomycin (9.1%), teicoplanin (9.1%), chloramphenicol (12.3%), and doxycycline (14.9%). The MDR isolates (43%, *n* = 67) showed diverse phenotypic relatedness. They showed multiple antibiotic resistance (MAR) index range from 0.31–1.0, exhibiting 100% non-susceptibility to cefoxitin (MRSA), erythromycin, and clarithromycin known as macrolide resistant *S. aureus* (McRSA), followed by 80%, 74.6%, and 46.2% for clindamycin, azithromycin, and PST, respectively. All the MDR isolates gave positive *nuc*, *mec*A and confirmed MRSA. The *erm*C, *erm*A, and *msr*A, genes were detected in 49.25%, 26.8%, and 23.8% of the MDR isolates, respectively. The PST-doxycycline and PST-levofloxacin combinations were mostly synergistic in 82.13% and 70.14%, while PST-linezolid showed mostly additive effects in 67% of the MDR *S. aureus* isolates.

**Conclusion:**

This study highlights the high prevalence of MRSA isolates recovered from various skin infections. Linezolid, vancomycin, teicoplanin, pristinamycin, chloramphenicol, and doxycycline remain effective therapeutic options. Macrolide and methicillin resistance are increasingly developing among *S. aureus* clinical isolates. The pristinamycin combination with doxycycline or levofloxacin was mostly synergistic and recommended for clinical evaluation.

**Supplementary Information:**

The online version contains supplementary material available at 10.1186/s12879-025-11433-0.

## Background

The most common bacterium that causes purulent skin and soft tissue infections is *Staphylococcus aureus* (*S. aureus*) [1]. It can cause a wide array of infections, from simple skin infections to potentially fatal bacteremia [2]. Staphylococcal skin infections are among the most frequent infections of both surgical sites and chronic wounds [2, 3]. *S. aureus* is the *Staphylococcus* species most usually associated with antibiotic resistance and included in the ESKAPE group, a group of the most important bacteria engaged in infections and defined by multidrug-resistant (MDR) pathogens [3]. Additionally, methicillin-resistant *S. aureus* (MRSA) is frequently the source of staphylococcal skin infections, which provide a therapeutic challenge and significantly slow down the healing process of wounds [4, 5]. Aggravating the problem of *S. aureus* infection is the emergence of virulent strains that are resistant to a wide range of antibiotics [6, 7]. For many years, MRSA infections have presented a clinical challenge. MRSA infections usually have higher rates of morbidity and mortality, which is linked to longer hospitalization and related costs as compared to those caused by methicillin-susceptible *S. aureus* (MSSA) [8, 9]. Resistance has rapidly emerged in response to the use of potential anti-staphylococcal medicines, especially against last-line medications like linezolid, daptomycin, and anti-MRSA cephalosporins [7]. Although vancomycin resistance is still uncommon, the emergence of vancomycin-intermediate *S. aureus* strains is alarming [10].

MRSA is among twelve deadly pathogenic species that the World Health Organization has nominated as high- to medium-priority-resistant bacteria that need to be treated right away [11]. One of the most effective classes of antibiotics, macrolide-lincosamide-streptogramin B (MLSB), was frequently prescribed to treat infections caused by those pathogens [12]. Streptogramin antibiotics that are produced by many species of *Streptomyces* encompass two structurally different subgroups: group A and group B [13, 14]. Separately, group A and B streptogramins are bacteriostatic because they bind reversibly to the 50S subunit of the bacterial ribosome. However, combined streptogramins including pristinamycin (PST), quinupristin + dalfopristin, and virginiamycin are synergic and bactericidal [15]. Furthermore, they have been employed to treat a wide variety of bacterial infections, including those caused by vancomycin-resistant *S. aureus* and vancomycin-resistant enterococci [16]. Pristinamycin (PST) is a naturally occurring streptogramin that was isolated from the *Streptomyces pristinaespiralis* bacteria [17]. It contains two synergistic components: PST IA (a macrolide-like streptogramin B-type); and PST IIA (a streptogramin A-type [18]. They bind to the 50S ribosomal subunit, resulting in suppression of protein synthesis and becoming bactericidal [19]. Staphylococcal resistance to synergistic mixtures is infrequently documented and is invariably related to resistance to the streptogramin A type (PST II A MICs of ≥ 8 mg/L), but not essentially resistance to the B type. Streptogramin resistance is uncommon among staphylococci [20]. However, the emergence of *S. aureus* strains resistant to PST was previously reported and imposed a real challenge in their treatment, for example, in Tunisia [20], India [21] and France [22]. With few available treatments, MRSA and macrolide-resistant *S. aureus* (McRSA) pose serious risks to human health, particularly those exhibiting non-susceptibility to PST [22]. In this study, different pristinamycin-antibiotic combinations were chosen for the following reasons: i) PST is not frequently used in our clinical setting, so we anticipated that it would retain better activity; ii) antimicrobial agents that, according to our findings, still retain activity against MRSA; iii) antibiotics that are less costly and commercially available in our clinical setting; and iv) those that are still pending evaluation. Moreover, few studies in the literature focus on investigating the antimicrobial activity of PST, either by itself or in combination with other antimicrobial agents. Therefore, this study aimed to examine the prevalence of the corresponding infections and their susceptibility to antibiotics, with particular emphasis on susceptibility to PST in one of the major tertiary care Hospitals in Egypt. In addition, this study aimed to study the effect of new PST-antibiotic combinations against McRSA and MRSA clinical isolates.

## Methods

### Study design and microbiological procedures

This study was performed over 12 months, starting from March 2022 to February 2023, et al.-Demerdash Hospital, Cairo, Egypt. Based on the hospital records, a total of 467 clinical specimens were received by the central microbiology lab for culture and sensitivity testing, including pus exudates from wounds, abscesses, skin burns, and surgical sites. Positive bacterial cultures were obtained from 276 specimens, of which 154 showed growth of *S. aureus* on mannitol salt agar*,* and they were selected for enrollment in this study. Standard biochemical tests, including catalase, coagulase, hemolysin, and gelatin hydrolysis, microbiological diagnostics, including growth on mannitol salt agar and colony morphology, and Gram staining, were routinely used to identify positive cultures according to Bergey’s manual of determinative bacteriology [23]. The automated system Vitek-2 (bioMérieux, Marcy L’Etoile, France) was used to confirm identification.

### Antimicrobial susceptibility and calculation of multiple antibiotic resistance (MAR) index

The recovered *S. aureus* isolates were tested for susceptibility to 22 different antimicrobial agents according to the Clinical and Laboratory Standards Institute (CLSI, 2021) [24]. The antimicrobial agents used were: penicillin (P, 10U), ampicillin (AM, 10 μg), amoxicillin/clavulanic acid (AMC, 20/10 μg), cefoxitin (FOX, 30 μg), gentamicin (CN, 10 μg) amikacin (AK, 30 μg), trimethoprim-sulfamethoxazole (COT, 1.25/23.75 μg), ciprofloxacin (CIP, 5 μg), levofloxacin (LEV, 5 μg), norfloxacin (NOR, 10 μg), ofloxacin (OFX, 5 μg), doxycycline (DO, 30 μg), erythromycin (E, 15 μg), clarithromycin (CLR, 15 μg), azithromycin (AZM, 15 μg), clindamycin (DA, 2 μg), chloramphenicol (C, 30 μg), vancomycin (V, 30 μg), rifampin (RF, 5 μg), linezolid (LNZ, 30 μg), teicoplanin (TEI, 30 μg) and nitrofurantoin (F, 300 μg). The PST susceptibility was quantitatively determined for the MDR *S. aureus* using the MIC by micro-broth dilution using the MIC values of quinupristin-dalfopristin (S ≤ 1.0; I = 2; R ≥ 4) [24]. The susceptibility of staphylococci to vancomycin was determined using E-Test (Bioanalyse, Turkey). The standard *S. aureus* strains ATCC 25923 and ATCC 29213 were used as a reference for the quality control of the disk diffusion and MIC assessment by broth dilution methods, respectively. The MDR phenotype was determined as formerly described by Magiorakos et al. [25]. The multiple antibiotic resistance (MAR) index was calculated as previously reported [26]. The MDR phenotype is defined as the isolate-acquired non-susceptibility to at least one agent in three or more antimicrobial categories.

### Molecular characterization of the collected MDR *S. aureus*

Molecular characterization of the collected MDR *S. aureus* was carried out by testing four clinically relevant resistant genes, including erythromycin ribosomal methylase type A (*erm*A), type C (*erm*C), MAC-streptogramin resistance (*msr*A), and antibiotic-resistant penicillin-binding protein PBP 2a, *mec*. PCR analysis of the *nuc* gene was done as a marker gene to molecularly identify the isolates. The primers used are displayed in Table 1, and the PCR conditions were performed as previously described [14, 27].

**Table 1:** The primer sequences used in this study and the expected PCR product sizes

Primer	Target gene	Primer sequence (5’−3’)	Annealing Temperature, °C (Ta)	PCR product size (bp)	Reference
*erm*A-F	*erm*A	TATCTTATCGTTGAGAAGGGATT	50	139	[14, 27]
*erm*A-R		CTACACTTGGCTTAGGATGAAA			
*erm*C-F	*erm*C	AATCGTCAATTCCTGCATGT	51	299	[14, 27]
*erm*C-R		TAATCGTGGAATACGGGTTTG			
*msr*A-F	*msr*A	TCCAATCATAGCACAAAATC	47	163	[14, 27]
*msr*A-R		AATTCCCTCTATTTGGTGGT			
*mec*A-F	*mec*A	AAAATCGATGGTAAAGGTTGGC	50	533	[14, 27]
*mec*A-R		AGTTCTGGAGTACCGGATTTGC			
*nuc*-F	*nuc*	GCGATTGATGGTGATACGGT	50	433	[14, 27]
n*uc*-R		AGCCAAGCCTTGACGAACTAAAGC			

*erm*A erythromycin 23S ribosomal methylase gene A, *erm*C erythromycin 23S ribosomal methylase gene C, *msr*A MAC-streptogramin efflux resistance gene, *mec*A encodes the protein PBP2A (penicillin-binding protein 2 A), *nuc* thermostable nuclease, *bp* base pair

### Evaluation of the phenotypic relatedness of MDR *S. aureus* isolates

Morpheus online software was used to perform the heatmap analysis to assess the phenotypic relatedness of the MDR isolates, including both McRSA and MRSA clinical isolates that were collected (https://software.broadinstitute.org/morpheus/) (accessed in January 2025) using Euclidean distances as previously described [28]. The heatmap analysis was conducted based on the antimicrobial susceptibility of collected MDR *S. aureus* isolates.

### Evaluation of five PST-antibiotic combinations against MDR *S. aureus* isolates

Five-antibiotic combinations of PST (Product No. SBR00044, Sigma-Aldrich, Jakarta, Indonesia) with either cefoxitin (FOX), linezolid (LZD), doxycycline (DOX), levofloxacin (LEV), gentamicin (CN) or doxycycline have been evaluated against the collected MDR *S. aureus* using the checkerboard assay [29]. Each drug's fraction inhibitory concentration (FIC) was evaluated as formerly reported [30]. The FIC of each antibiotic was calculated by dividing its MIC in combination with its MIC when tested separately. The ΣFIC index is the sum of the FIC of both antibiotics and is interpreted as: ≤ 0.5(synergism), > 0.5–1 (additive), > 1–4.0 (indifference), and > 4 (antagonism) [29, 30].

### Statistical analysis

Microsoft EXCEL Office 365 was used to construct figures and tables and calculate the frequency of the isolates and their percentage of resistance.

## Results

### Bacterial pathogens

A total of 467 clinical specimens were received by the central microbiology lab for culture and sensitivity testing, including pus exudates from wounds, abscesses, skin burns, and surgical sites. Positive bacterial cultures were obtained from 276 specimens, of which 154 showed growth of *S. aureus* on mannitol salt agar*,* and they were selected for enrollment in this study. The mean age of the enrolled participants (*n* = 154) showing positive *S. aureus* culture was 41.2 ± 14.5 years; of these 66.23% (*n* = 102) were Male, 33.76% (*n* = 52) were female. These patients were febrile neutrophilic (> 11,000 leucocyte count/µL with oral temperature > 38⁰C, for at least 60 min) according to hospital records. A total of 154 (55.97%) positive bacterial cultures for *S. aureus* were obtained from 276 clinical specimens, including wound (63; 40.9%), abscess (57; 37.0%), burn (22; 14.3%), and surgical site (12; 7.8%). The 154 *S. aureus* were examined for antimicrobial susceptibility against 22 antimicrobial agents.

### Antimicrobial susceptibility

**Fig. 1:**
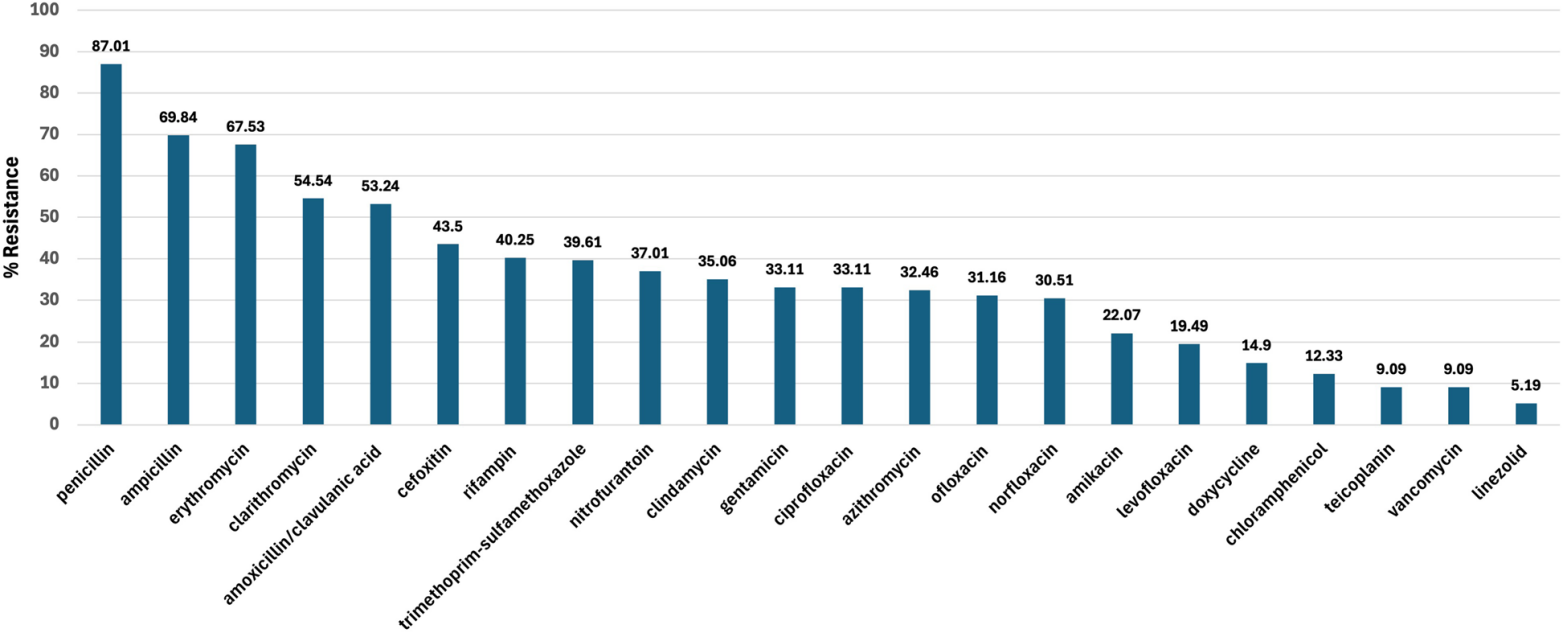
Antimicrobial susceptibility testing of the collected *S. aureus* (*n* = 154) from various clinical specimens against 22 different antimicrobial agents

As depicted in Fig. 1, the antimicrobial susceptibility of *S. aureus* isolates (*n* = 154) revealed that the highest resistance was detected against penicillin (134; 87%), followed by ampicillin (107; 70%) and erythromycin (104; 67.5%). The cefoxitin resistance (suspected MRSA isolates) was detected in 43.5% (*n* = 67) of the tested isolates. The percentage of McRSA was detected to be in the range of 32.46–67.5% against the tested macrolides, including azithromycin (32.46%), clarithromycin (54.54%), erythromycin (67.53%) and to the lincosamide antibiotic clindamycin (35.06%), On the contrary, the lowest antimicrobial resistance of the collected *S. aureus* isolates was detected against linezolid (8, 5.2%), followed by vancomycin (14; 9.1%), teicoplanin (14; 9.1%), chloramphenicol (19; 12.3%) and doxycycline (23; 14.9%). The percentage of *S. aureus* clinical isolates that exhibited MDR phenotype was 43.5% (*n* = 67).

### Antimicrobial susceptibility of MDR isolates and MAR index calculation

**Fig. 2:**
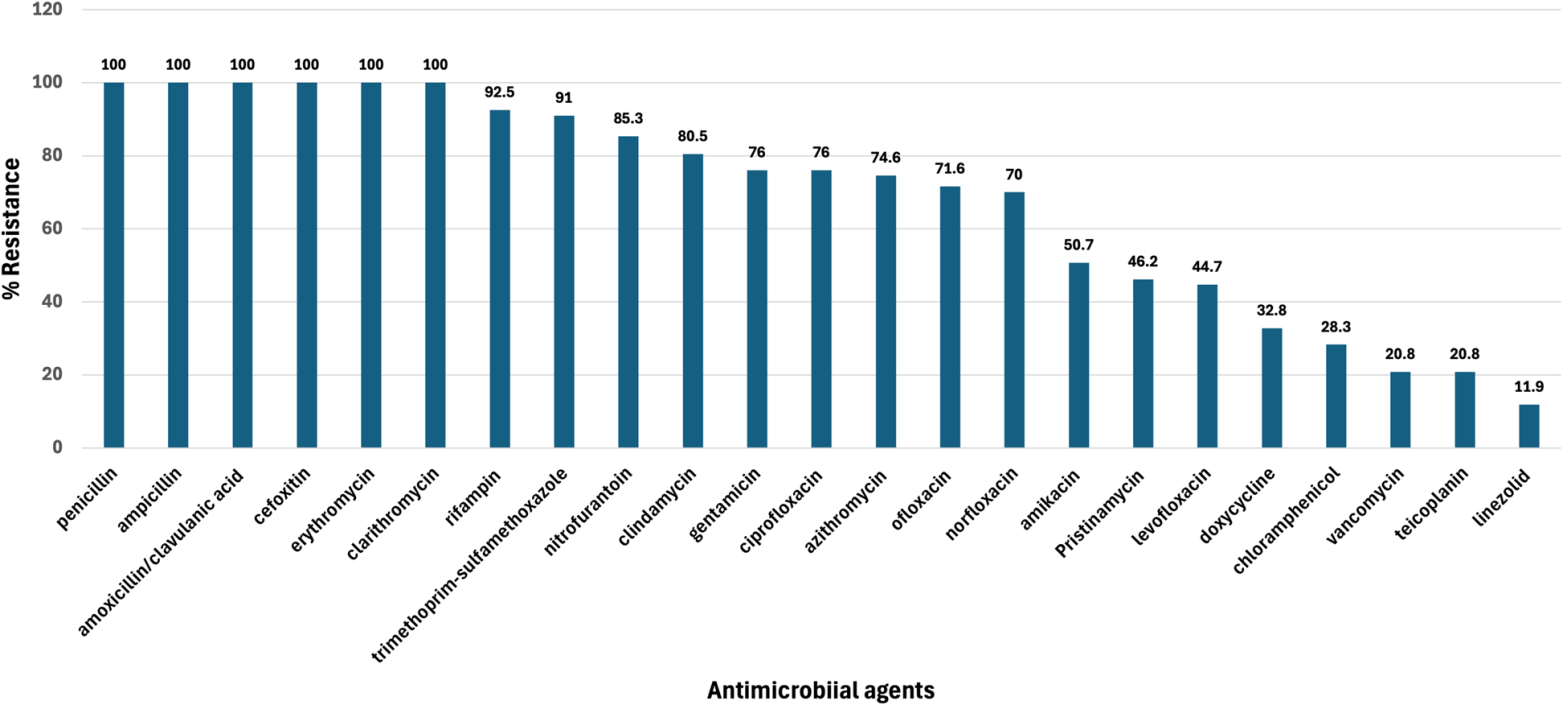
Antimicrobial susceptibility testing of the collected multidrug-resistant (MDR) *S. aureus* (*n* = 67) from various clinical specimens against 23 different antimicrobial agents

As displayed in Fig. 2, the antimicrobial susceptibility of MDR *S. aureus* clinical isolates (*n* = 67) is displayed. Results showed that 100% (*n* = 67) exhibited cefoxitin resistance and showed 100% resistance to other penicillins, including ampicillin and amoxicillin-clavulanate. For Macrolides, they showed 100% (*n* = 67) resistance to erythromycin and clarithromycin and 80% (*n* = 54) and 74.6% (*n* = 50) for clindamycin and azithromycin, respectively. They also exhibited 46.2% (*n* = 31) resistance to PST. Therefore, 100% of the MDR isolates (*n* = 67) exhibited both macrolide resistance (McRSA) and cefoxitin resistance. The lowest resistance was observed for linezolid (8, 11.9%), followed by vancomycin (14; 14.9%), teicoplanin (14; 14.9%), chloramphenicol (19; 28.4%) and doxycycline (22; 32.8%). The detailed antimicrobial susceptibility of MDR isolates against the tested is displayed in Table S1. The results of the MAR index of the MDR *S. aureus* (*n* = 67) are displayed in Table S1, where all isolates exhibited different MAR patterns (MaR ranged from 0.31 to 1.0. Results revealed that two isolates (2.98%) exhibited MAR = 1.0, conferring pan-drug-resistance (PDR) phenotypes, 17 isolates (25.3%) showed MaR index from 0.81–9.5, 41 isolates (61.2%) exhibited MaR range from 0.5–0.77, and 7 isolates (10.4%) showed MaR range from 0.31–0.45.

### Heatmap analysis of phenotypic relatedness

**Fig. 3:**
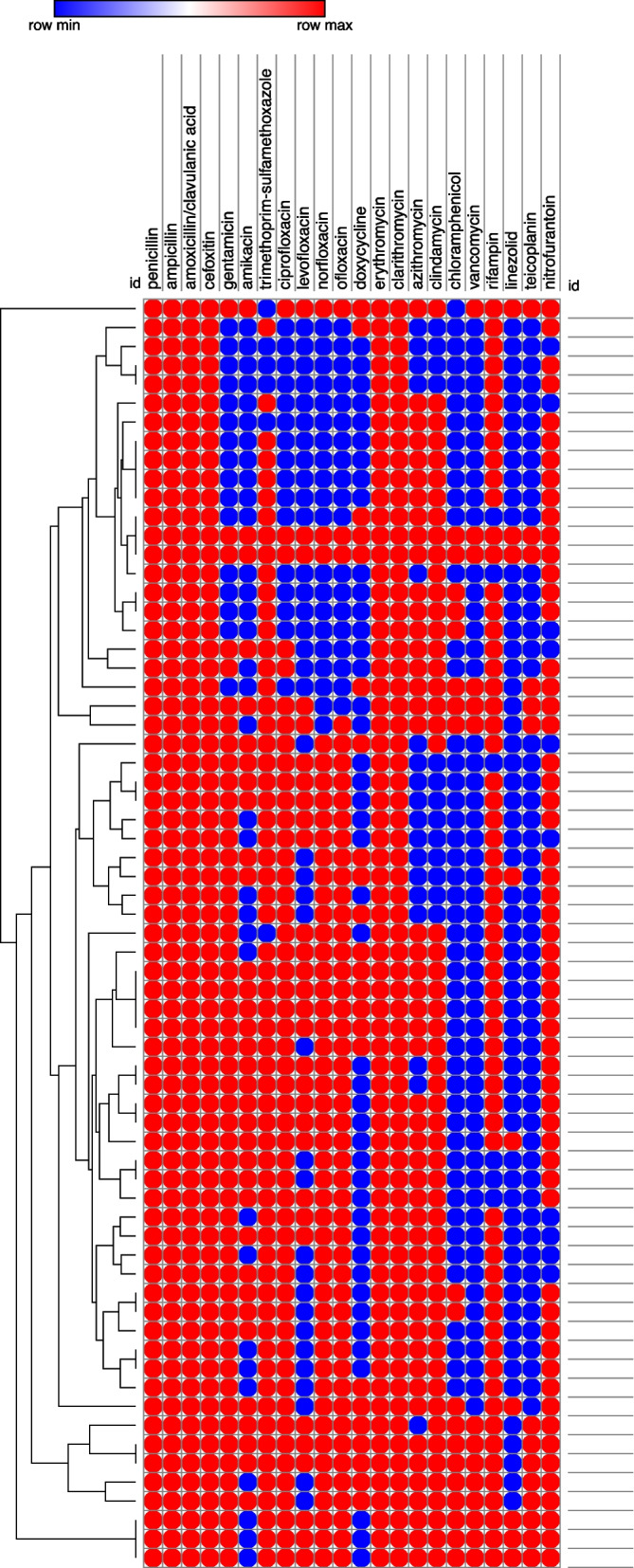
Heatmap analysis for evaluating the phenotypic relatedness of the collected multidrug-resistant (MDR) *S. aureus* (*n* = 67). The red circle indicates resistance to the tested antimicrobial agents; the Blue circle indicates sensitivity to the tested antimicrobial agents

Figure 3 delineates the results of the phenotypic analysis of the MDR *S. aureus* isolates using Euclidean distances. The results showed that the collected MDR isolates (*n* = 67) were aligned in 47 groups, indicating their diversity and non-clonal relatedness.

### Molecular characterization using PCR

**Fig. 4:**
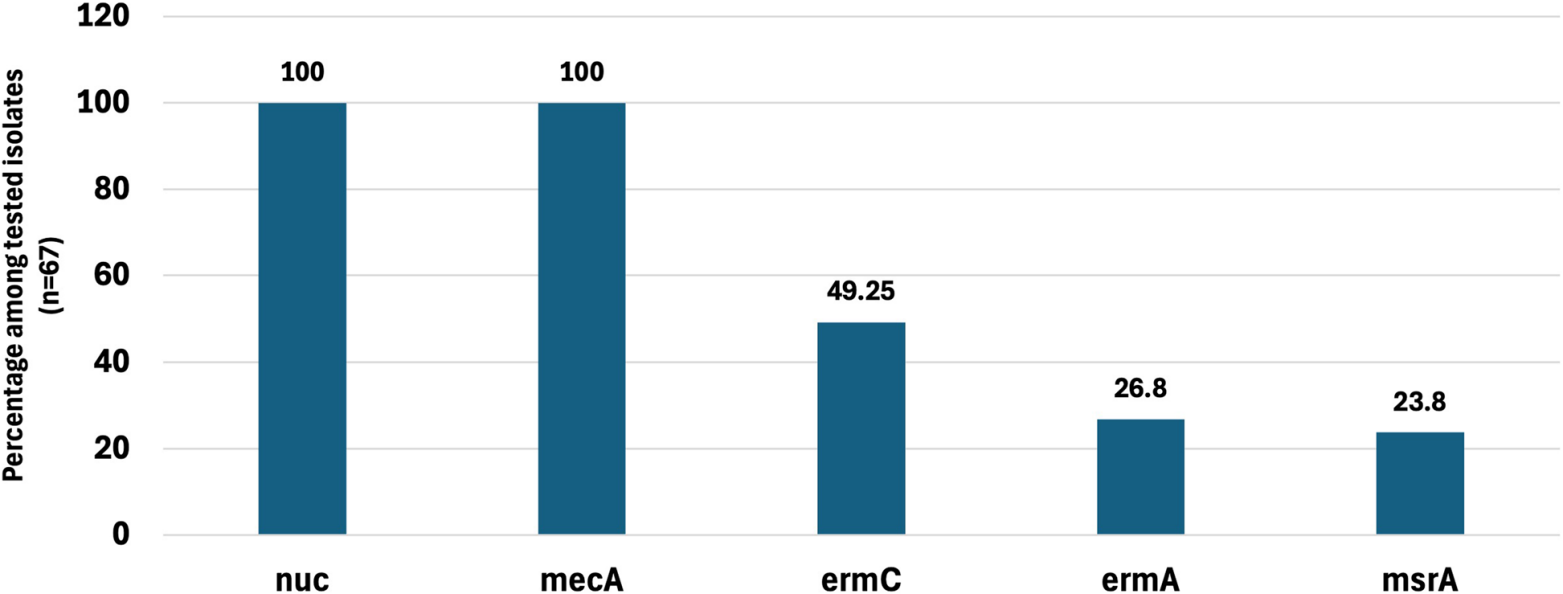
Molecular characterization of the collected MDR *S. aureus* (*n* = 67). *erm*A, erythromycin 23S ribosomal methylase gene A; *erm*C, erythromycin 23S ribosomal methylase gene C; *msr*A, MAC-streptogramin efflux resistance gene; *mec*A, encodes the protein PBP2A (penicillin-binding protein 2 A); *nuc,* thermostable nuclease

Since the MDR *S. aureus* isolates (*n* = 67) exhibited 100% resistance to erythromycin, clarithromycin (McRSA), and cefoxitin resistance (MRSA), they were examined for the four resistance genes (*erm*A, *erm*C, *msr*A, and *mec*A) and the *nuc* gene as a marker of identification of *S. aureus* using PCR. The genotypic profile of the collected isolates is displayed in Table S2. The percentage of the detected genes was as follows: 100%, 100%, 49.25%, 26.8% and 23.8% for *nuc, mecA, ermC, ermA*, and *msr*A, respectively, as depicted in Fig. 4. The presence of *nuc* gene in all isolates confirmed that all 67 isolates were *S. aureus*. The *mec*A gene was detected in the 67 isolates (100%), confirming all isolates were MRSA. The *erm*C coded for 23S ribosomal methylase gene type C (33; 49.25%) was the most prevalent macrolide-resistant gene, followed by *erm*A (18; 26.8%) coded for 23S ribosomal methylase gene A and *msr*A (16; 23.8%) coded for the MAC-streptogramin efflux resistance gene (Fig. 4).

### Evaluation of five pristinamycin (PST)-antibiotic combinations against McRS and MRSA isolates

**Fig. 5:**
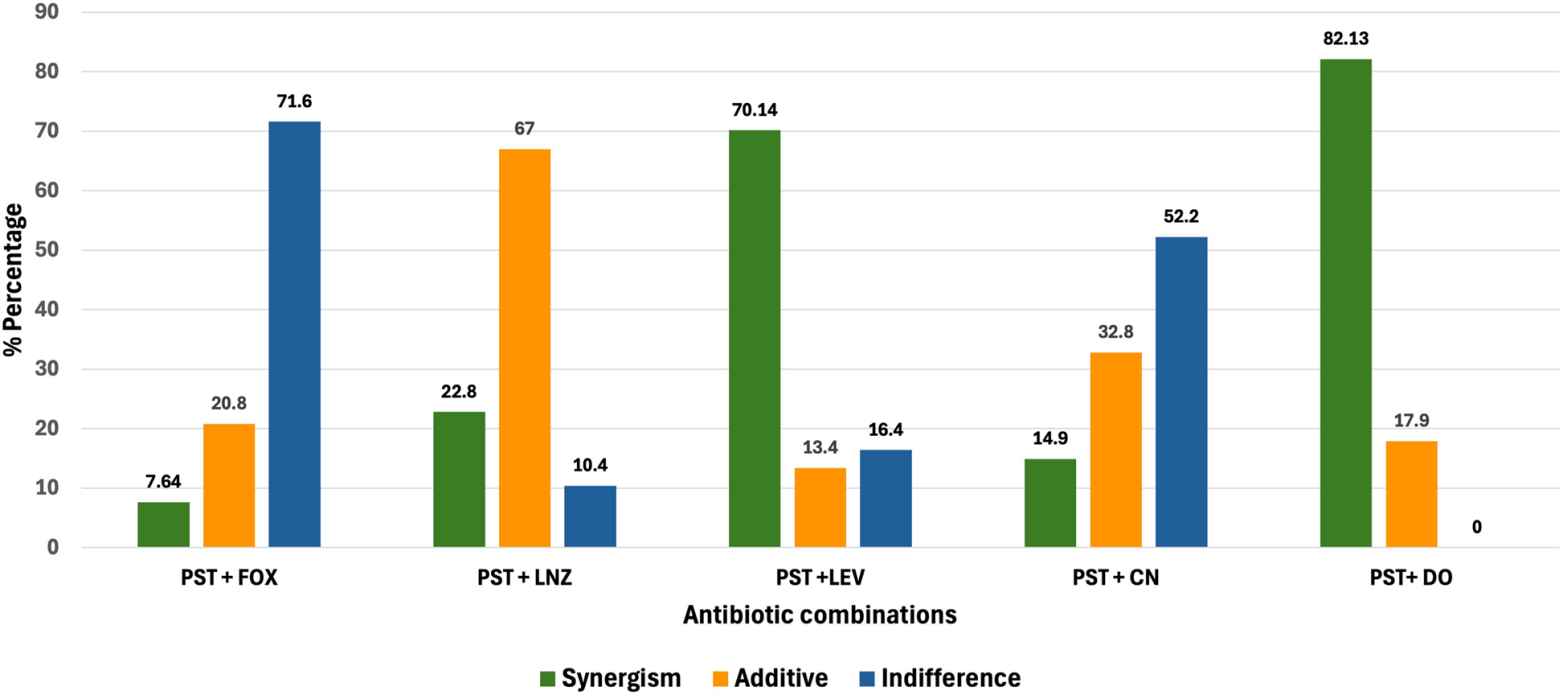
Evaluation of five PST-antibiotic combinations against MDR *S. aureus* isolates (*n* = 67). PST, pristinamycin; FOX, cefoxitin; LNZ, linezolid; LEV, levofloxacin, CN, gentamicin; DO, doxycycline

As depicted in Fig. 5, pristinamycin-doxycycline (PST-DO) and pristinamycin-levofloxacin (PST-LEV) showed 82.13% and 70.14% synergism among the tested MDR *S. aureus* (*n* = 67), respectively. The pristinamycin (PST) combination with linezolid (LNZ) showed mostly additive effects (67%) on the tested MDR isolates. The pristinamycin-cefoxitin (PST-FOX) and pristinamycin-gentamicin (PST-CN) showed mostly indifference in 71.6% and 52.2% of the tested MDR. *S. aureus* isolates. The detailed fractional inhibitory concentration (FIC) values of the five antibiotic combinations are shown in Table S3.

## Discussion

With few available treatments, methicillin-resistant and macrolide-resistant *S. aureus* (McRSA) pose serious risks to human health and are characterized by limited therapeutic options [14, 31]. This study aimed to identify the respective pathogens'prevalence and susceptibility profile to various antibiotics being employed in their treatment in one of the Major tertiary care Hospitals in Egypt. In addition, we aimed to evaluate the antimicrobial activity of PST alone and in combination with other commonly used antimicrobial agents in treating McRSA and MRSA in Egypt. In the literature, limited studies are available that are concerned with examining the antimicrobial activity of PST either alone or as combination with other antimicrobial agents. In this study, a total of 154 non-clonal *S. aureus* clinical isolates were collected during the period from March 2022 to February 2023 and were evaluated against 22 antimicrobial agents according to CLSI guidelines [24]. Results showed that about 43% of the collected isolates were MDR and the highest non-acceptability was recorded against beta-lactams (70–87%), including cefoxitin (43.5%), penicillin (87%) as well as macrolides including erythromycin (67.5%), azithromycin (32.46%), clindamycin (35.06%), and clarithromycin (54.54%). However, the lowest resistance was noted against linezolid (5.2%), which was followed by doxycycline (14.9%), vancomycin (9.1%), teicoplanin (9.1%), and chloramphenicol (12.3%). Our findings are in alignment with the findings of other recent studies [32–35]. Based on these findings, further investigations are urgently required to find the solution to combat the rise in antimicrobial non-susceptibility, particularly toward beta-lactams and macrolides, being developed by *S. aureus* clinical isolates. A few years after macrolides were introduced into the treatment, the first staphylococcal strains resistant to these antibiotics emerged. Currently, MAC resistance is common globally, and many bacteria are resistant to MACs, lincosamides, and streptogramin type B (MLSB) antibiotics [36]. The rising MAC resistance among staphylococci could be attributed to their frequent use in our clinical setting, which is usually coupled with the resistance to lincosamides and streptogramins as previously reported [31].

As previously reported, there are three primary mechanisms of MLSB resistance in staphylococci, including target site modification, efflux of MAC outside the cell, and enzymatic inactivation. Nevertheless, only the first two play a significant role in *S. aureus* resistance [37]. Because the MDR *S. aureus* isolates exhibited 100% resistance to erythromycin, clarithromycin, and cefoxitin, they were selected for testing three of the most common MAC-resistant genes. Four clinically relevant resistant genes were tested, including erythromycin ribosomal methylase type A (*erm*A), type C (*erm*C), MAC-streptogramin resistance (*msr*A), to identify the macrolide resistance genes that could be involved in MAC resistance [14]. The antibiotic-resistant penicillin-binding protein PBP 2a, *mec*A, was tested to confirm MRSA phenotype, however, the specific thermo-nuclease (*nuc*) as a virulence gene was tested to confirm the pathogenicity and identity of the recovered *S. aureus* [27]. For the MDR *S. aureus* clinical isolates recovered in this study, results showed that all the detected MDR isolates (100%) were resistant to cefoxitin resistance, ampicillin, and amoxicillin-clavulanate, erythromycin, and clarithromycin. In addition to that, 80%, 74.6%, and 46.2% exhibited non-susceptibility to clindamycin, azithromycin, and PST, respectively. The lowest resistance was observed for linezolid (11.9%), followed by vancomycin (14.9%), teicoplanin (14; 14.9%), chloramphenicol (28.4%), and doxycycline (32.8%). According to the MDR *S. aureus* MAR index results (*n* = 67), each isolate had a unique MaR pattern, with MaR ranging from 0.31 to 1.0. The findings showed that two isolates (90.9%) had pan-drug-resistance (PDR) phenotypes due to MAR = 1.0, while 17 isolates (25.3%) had MaR indexes between 0.81 and 9.5, 41 isolates (61.2%) had MaR ranges between 0.5–0.77, and 7 isolates (10.4%) had MaR ranges between 0.31 and 0.45. The respective findings indicated a high level of antimicrobial resistance of the MDR isolates as compared to previous reports [38–40]. Therefore, further molecular analysis of the major antibiotic resistance genes was important to correlate antibiotic resistance to the potentially identified genetic determinants responsible for such resistance.

Molecular characterization revealed that all the MDR isolates (*n* = 67) gave 100% positive PCR for the *nuc* gene and *mec*A gene confirming that all isolates exhibited MRSA phenotypes. Regarding the MAC-resistant genes, the *erm*C (49.25%) was the most prevalent MAC-resistant gene, followed by the *erm*A (26.8%) and the *msr*A gene (23.8%). Our findings are in accordance with previous studies regarding the prevalence of the respective MAC-resistant genes among *S. aureus* clinical isolates [14, 35–37].

These findings initiated a further investigation to evaluate five antibiotic combinations of PST with each of the antimicrobial agents, FOX, LNZ, LEV, CN, and DO. The rationale for using these combinations was: i) PST is not commonly used in our clinical setting, so we expected to retain a better activity; ii) antimicrobial agents that still retain activity against MDR *S. aureus* based on our findings; iii) agents that are less expensive and commercially available in our clinical setting; and iv) those still not being evaluated. The synergistic/additive activities of certain combinations could be achieved regardless of whether the isolate is resistant to one of the tested antibiotics since it was based on the calculation of the ΣFIC index, which is based on the values of the MIC of each antibiotic alone and in combination. Our Findings showed that the studied MDR *S. aureus* (*n* = 67) exhibited 82.13% and 70.14% synergism with pristinamycin-doxycycline (PST-DO) and pristinamycin-levofloxacin (PST-LEV), respectively. However, they exhibited primarily additive effects (67%) when pristinamycin (PST) and linezolid (LNZ) were combined. In 71.6% and 52.2% of the tested MDR *S. aureus* isolates, respectively, pristinamycin-cefoxitin (PST-FOX) and pristinamycin-gentamicin (PST-CN) exhibited largely indifference.

Our study was focused on the use of PST as a new member of streptogramins and evaluated it either alone or as a combination of the other five antibiotics that are usually employed for the treatment of various infections caused by *S. aureus*. This is due to streptogramins'quick bactericidal action against a variety of pathogens, low incidence of resistance in clinical isolates, and favorable pharmacokinetic characteristics, which have made them viable substitutes for the treatment of Gram-positive infections [41]. PST looks to be a well-tolerated, effective oral option for treating tough Gram-positive infections, such as VRE and MRSA, in a variety of difficult-to-treat or protected-site illnesses [42]. Streptogramins, particularly PST, are viable and effective alternatives for the treatment of Gram-positive infections [41]. Oral treatment options for MDR *S. aureus* infections are limited. Pristinamycin (PST) is a possible alternative and has promising efficacy either alone or in combination with other antibiotics [43]. It was reported by Cocito et al*.* that MLSB phenotype alone is not sufficient to generate PST resistance as a variety of other resistance mechanisms is necessary to achieve high-level resistance to PST [44]. Despite 55% of isolates being resistant to lincosamides or having inducible resistance, Dancer et al. also found an 87% success rate in treating MRSA infections with PST, primarily affecting the skin and soft tissues, suggesting MLSB phenotype [45]. Similarly, Reid et al. treated 26 patients with primarily osteoarticular infections caused by 31 different staphylococci with PST. They found that eight were cured, 15 were suppressed, and 3 failed therapies because of drug intolerance, not uncontrolled illness [42]. Moreover, the in vitro MLSB phenotype of macrolide and lincosamide resistance does not appear to be related to PST resistance. Similarly, clinical investigations suggest satisfactory outcomes using PST in suspected MLSB staphylococcal infections [44]. Our findings showed that PST is the MAC antibiotic that exhibited the highest activity against MDR *S. aureus*. In addition, PST combination with doxycycline or levofloxacin achieved the highest synergism. These antibiotic combinations as highly recommended for clinical evaluation for potential use in humans against McRSA and MRSA clinical isolates.

## Conclusion

This study highlights the rise in the prevalence of the MDR *S. aureus* phenotype, including the MAC-resistant and MRSA isolates that were recovered from various types of skin infections. Pristinamycin, doxycycline, linezolid, vancomycin, and teicoplanin still retain good activity. However, resistance to the respective antibiotics developed among clinical isolates of *S. aureus* in clinical settings, and therefore, new guidelines should be undertaken to combat such resistance and for infection control. The PST combination with levofloxacin or doxycycline was primarily synergistic; however, its combination with linezolid showed mostly additive effects. This is the first report confirming the synergy of pristinamycin combination with doxycycline or levofloxacin against MRSA recovered from skin infections.

## Supplementary Information

Supplementary Material 1.

## Data Availability

All data generated or analyzed during this study are included in this published article and supplementary file. The nucleotide sequences of msrA gene was deposited in the NCBI GenBank database under the accession code, KJ710361, (https://www.ncbi.nlm.nih.gov/nuccore/KJ710361.1), mecA gene under the accession code, MK341125, https://www.ncbi.nlm.nih.gov/nuccore/MK341125.1.
