# Supplementary material for: Pristinamycin-antibiotic combinations against methicillin-resistant Staphylococcus aureus recovered from skin infections
Source: BMC Infect Dis. 2025 Sep 16;25:1107. doi: 10.1186/s12879-025-11433-0 (PMC12439405; doi:10.1186/s12879-025-11433-0)
Supplement: Supplementary file 1 — Supplementary Material 1. [file 12879_2025_11433_MOESM1_ESM.docx]

**Table S1.** Antimicrobial susceptibility testing and multiple antibiotic resistance (MAR) index of the MDR *S. aureus* clinical isolates (n=67)

| **Isolate** | **MAR index** | **P** | **AMP** | **AMC** | **FOX** | **CN** | **AK** | **COT** | **CIP** | **LEV** | **NOR** | **OFX** | **DO** | **E** | **CLR** | **AZM** | **DA** | **C** | **V** | **RF** | **LNZ** | **TEI** | **NIT** |
| --- | --- | --- | --- | --- | --- | --- | --- | --- | --- | --- | --- | --- | --- | --- | --- | --- | --- | --- | --- | --- | --- | --- | --- |
| SA-1 | 0.5 | R | R | R | R | S | S | R | S | S | S | S | R | R | R | R | R | S | S | S | S | S | R |
| SA-4 | 0.45 | R | R | R | R | S | S | R | S | S | S | S | R | R | R | S | S | S | S | R | S | S | R |
| SA-6 | 0.68 | R | R | R | R | R | R | R | R | S | R | R | R | R | R | S | S | S | S | R | S | S | R |
| SA-8 | 0.90 | R | R | R | R | R | S | R | R | R | R | R | S | R | R | R | R | R | R | R | R | R | R |
| SA-12 | 0.77 | R | R | R | R | R | S | R | R | R | R | R | R | R | R | R | R | S | S | R | S | S | R |
| SA-17 | 0.68 | R | R | R | R | R | R | R | R | S | R | R | R | R | R | S | R | S | S | R | S | S | S |
| SA-22 | 0.45 | R | R | R | R | S | S | S | S | S | S | S | S | R | R | R | R | S | S | R | S | S | R |
| SA-23 | 0.64 | R | R | R | R | R | R | R | R | R | R | R | S | R | R | S | S | S | S | S | S | S | R |
| SA-25 | 0.68 | R | R | R | R | R | S | S | R | R | R | R | S | R | R | R | R | S | S | R | S | S | R |
| SA-33 | 0.90 | R | R | R | R | R | R | R | R | R | R | R | R | R | R | S | R | R | R | R | S | R | R |
| SA-34 | 0.54 | R | R | R | R | S | S | R | S | S | S | S | S | R | R | R | R | R | S | R | S | S | R |
| SA-36 | 0.31 | R | R | R | R | S | S | S | S | S | S | S | S | R | R | S | S | S | S | R | S | S | S |
| SA-38 | 0.81 | R | R | R | R | R | R | R | R | R | R | R | R | R | R | R | R | S | S | R | S | S | R |
| SA-39 | 0.72 | R | R | R | R | R | R | R | R | R | R | R | S | R | R | S | R | S | S | R | S | S | R |
| SA-41 | 0.50 | R | R | R | R | S | S | R | S | S | S | S | S | R | R | R | R | S | S | R | S | S | R |
| SA-42 | 0.45 | R | R | R | R | S | S | R | S | S | S | S | S | R | R | R | R | S | S | R | S | S | S |
| SA-44 | 0.90 | R | R | R | R | R | R | S | R | R | R | R | R | R | R | R | R | S | R | R | R | R | R |
| SA-45 | 0.50 | R | R | R | R | S | S | R | S | S | S | S | S | R | R | R | R | S | S | R | S | S | R |
| SA-47 | 0.36 | R | R | R | R | S | S | S | S | S | S | S | S | R | R | S | S | S | S | R | S | S | R |
| SA-48 | 0.40 | R | R | R | R | S | S | R | S | S | S | S | S | R | R | S | R | S | S | S | S | S | R |
| SA-50 | 0.82 | R | R | R | R | R | R | R | R | R | R | R | R | R | R | R | R | S | S | R | S | S | R |
| SA-51 | 0.54 | R | R | R | R | S | S | R | S | S | S | S | S | R | R | R | R | R | S | R | S | S | R |
| SA-52 | 1.0 | R | R | R | R | R | R | R | R | R | R | R | R | R | R | R | R | R | R | R | R | R | R |
| SA-54 | 0.50 | R | R | R | R | S | S | R | S | S | S | S | S | R | R | R | R | S | S | R | S | S | R |
| SA-55 | 0.36 | R | R | R | R | S | S | S | S | S | S | S | S | R | R | S | S | S | S | R | S | S | R |
| SA-57 | 0.81 | R | R | R | R | R | R | R | R | R | R | R | R | R | R | R | R | S | S | R | S | S | R |
| SA-58 | 0.50 | R | R | R | R | S | S | R | S | S | S | S | S | R | R | R | R | S | S | R | S | S | R |
| SA-59 | 0.81 | R | R | R | R | R | R | R | R | R | R | R | R | R | R | R | R | S | S | R | S | S | R |
| SA-61 | 1.0 | R | R | R | R | R | R | R | R | R | R | R | R | R | R | R | R | R | R | R | R | R | R |
| SA-62 | 0.68 | R | R | R | R | S | S | R | S | S | S | S | R | R | R | R | R | R | R | R | S | R | R |
| SA-63 | 0.69 | R | R | R | R | R | S | R | R | R | R | R | S | R | R | R | R | S | S | R | S | S | S |
| SA-64 | 0.63 | R | R | R | R | R | S | R | R | R | R | R | S | R | R | S | S | S | S | R | S | S | R |
| SA-66 | 0.72 | R | R | R | R | R | R | R | R | R | R | R | S | R | R | S | R | S | S | R | S | S | R |
| SA-67 | 0.50 | R | R | R | R | S | S | R | S | S | S | S | S | R | R | R | R | R | S | R | S | S | S |
| SA-69 | 0.68 | R | R | R | R | R | R | R | R | R | R | R | S | R | R | S | S | S | S | R | S | S | R |
| SA-70 | 0.68 | R | R | R | R | R | R | R | R | S | R | R | S | R | R | R | R | S | S | S | S | S | R |
| SA-73 | 0.68 | R | R | R | R | R | R | R | R | R | R | R | S | R | R | S | S | S | S | R | S | S | R |
| SA-75 | 0.90 | R | R | R | R | R | S | R | R | R | R | R | S | R | R | R | R | R | R | R | R | R | R |
| SA-77 | 0.77 | R | R | R | R | R | R | R | R | S | R | R | S | R | R | R | R | R | S | R | S | S | R |
| SA-78 | 0.59 | R | R | R | R | R | R | R | R | S | S | S | S | R | R | R | R | S | S | R | S | S | S |
| SA-79 | 0.81 | R | R | R | R | R | R | R | R | R | R | R | S | R | R | R | R | S | S | R | R | S | R |
| SA-80 | 0.72 | R | R | R | R | R | R | R | R | R | R | R | S | R | R | R | R | S | S | R | S | S | S |
| SA-81 | 0.81 | R | R | R | R | R | R | R | R | R | S | S | S | R | R | R | R | R | R | R | S | R | R |
| SA-82 | 0.86 | R | R | R | R | R | S | R | R | S | R | R | R | R | R | R | R | R | R | R | S | R | R |
| SA-84 | 0.77 | R | R | R | R | R | R | R | R | R | R | R | S | R | R | R | R | S | S | R | S | S | R |
| SA-87 | 0.59 | R | R | R | R | R | S | R | R | S | S | S | S | R | R | R | R | S | S | R | S | S | R |
| SA-88 | 0.68 | R | R | R | R | R | R | R | R | S | R | R | S | R | R | R | R | S | S | S | S | S | R |
| SA-89 | 0.72 | R | R | R | R | R | R | R | R | R | R | R | S | R | R | R | R | S | S | S | S | S | R |
| SA-95 | 0.68 | R | R | R | R | R | S | R | R | S | R | R | S | R | R | R | R | S | S | R | S | S | R |
| SA-99 | 0.77 | R | R | R | R | R | R | R | R | S | R | R | S | R | R | R | R | R | S | R | S | S | R |
| SA-101 | 0.95 | R | R | R | R | R | R | R | R | R | R | R | R | R | R | R | R | R | R | R | S | R | R |
| SA-107 | 0.72 | R | R | R | R | R | R | R | R | S | R | R | S | R | R | R | R | S | S | R | S | S | R |
| SA-114 | 0.68 | R | R | R | R | R | S | R | R | S | R | R | S | R | R | R | R | S | S | R | S | S | R |
| SA-115 | 0.63 | R | R | R | R | R | S | R | R | S | R | R | S | R | R | R | R | S | S | R | S | S | S |
| SA-118 | 0.86 | R | R | R | R | R | R | R | R | S | R | R | R | R | R | R | R | R | S | R | R | S | R |
| SA-120 | 0.59 | R | R | R | R | R | S | R | R | S | R | R | S | R | R | S | S | S | S | R | S | S | R |
| SA-122 | 0.68 | R | R | R | R | R | R | R | R | S | R | R | S | R | R | R | R | S | S | R | S | S | S |
| SA-127 | 0.72 | R | R | R | R | R | S | R | R | S | R | R | R | R | R | R | R | S | S | R | S | S | R |
| SA-129 | 0.81 | R | R | R | R | R | S | R | R | R | S | R | S | R | R | R | R | R | R | R | S | R | R |
| SA-132 | 0.95 | R | R | R | R | R | R | R | R | R | R | R | R | R | R | R | R | R | R | R | S | R | R |
| SA-134 | 0.77 | R | R | R | R | R | R | R | R | S | R | R | R | R | R | R | R | S | S | R | S | S | R |
| SA-139 | 0.90 | R | R | R | R | R | R | R | R | S | R | R | R | R | R | R | R | R | R | R | S | R | R |
| SA-142 | 0.59 | R | R | R | R | R | S | R | R | R | R | R | S | R | R | S | S | S | S | R | S | S | S |
| SA-144 | 0.63 | R | R | R | R | R | S | R | R | S | R | R | R | R | R | S | S | S | S | R | S | S | R |
| SA-148 | 0.77 | R | R | R | R | R | R | R | R | R | R | R | S | R | R | R | R | S | S | R | S | S | R |
| SA-151 | 0.72 | R | R | R | R | R | R | R | R | S | R | R | R | R | R | S | S | S | S | R | R | S | R |
| SA-153 | 0.95 | R | R | R | R | R | S | R | R | R | R | R | S | R | R | R | R | R | R | R | R | R | R |

Penicillin (P), ampicillin (AM, oxacillin (OX), amoxicillin/clavulanic acid (AMC), cefoxitin (FOX), gentamicin (CN), amikacin (AK), trimethoprim-sulfamethoxazole (COT), ciprofloxacin (CIP), levofloxacin (LEV), norfloxacin (NOR), ofloxacin (OFX), doxycycline (DO), erythromycin (E), clarithromycin (CLR), azithromycin (AZM), chloramphenicol (C), clindamycin (DA,), vancomycin (V), rifampin (RF), linezolid (LNZ), and teicoplanin (TEI), nitrofurantoin (NIT).

**Table S2.** Genotypic analysis of the MDR *S. aureus* clinical isolates (n=67)

| **Isolate** | **Detected virulence genes** | ***nuc*-gene** | **Isolate** | **Detected virulence genes** | ***nuc*-gene** | **Isolate** | **Detected virulence genes** | ***nuc*-gene** |
| --- | --- | --- | --- | --- | --- | --- | --- | --- |
| SA-1 | *ermA, ermC, mecA* | *+* | SA-55 | *mecA* | *+* | SA-95 | *ermC, mecA* | *+* |
| SA-4 | *mecA* | *+* | SA-57 | *mecA, ermC, msrA* | *+* | SA-99 | *ermC, mecA* | *+* |
| SA-6 | *mecA* | *+* | SA-58 | *ermC, mecA* | *+* | SA-101 | *mecA, msrA* | *+* |
| SA-8 | *ermA, ermC, msrA, mecA* | *+* | SA-59 | *mecA, ermC* | *+* | SA-107 | *ermC, msrA, mecA* | *+* |
| SA-12 | *ermA, mecA* | *+* | SA-61 | *ermC, mecA* | *+* | SA-114 | *ermC, mecA* | *+* |
| SA-17 | *ermA, mecA* | *+* | SA-62 | *ermC, mecA* | *+* | SA-115 | *ermC , mecA* | *+* |
| SA-22 | *ermA, mecA, msrA* | *+* | SA-63 | *ermA, mecA, msrA* | *+* | SA-118 | *ermC, mecA* | *+* |
| SA-23 | *mecA* | *+* | SA-64 | *mecA* | *+* | SA-120 | *mecA* | *+* |
| SA-25 | *ermA, mecA, msrA* | *+* | SA-66 | *ermC, mecA* | *+* | SA-122 | *ermC, mecA* | *+* |
| SA-33 | *ermC, mecA* | *+* | SA-67 | *ermC, mecA* | *+* | SA-127 | *ermA, mecA* | *+* |
| SA-34 | *ermA, mecA* | *+* | SA-69 | *mecA* | *+* | SA-129 | *ermC, mecA* | *+* |
| SA-36 | *mecA* | *+* | SA-70 | *ermC, mecA* | *+* | SA-132 | *mecA* | *+* |
| SA-38 | *mecA, msrA* | *+* | SA-73 | *mecA* | *+* | SA-134 | *ermA, ermC, mecA* | *+* |
| SA-39 | *ermA, mecA* | *+* | SA-75 | *mecA, msrA* | *+* | SA-139 | *mecA ermC, msrA* | *+* |
| SA-41 | *ermA, mecA* | *+* | SA-77 | *mecA, ermC, msrA* | *+* | SA-142 | *mecA* | *+* |
| SA-42 | *ermC, mecA* | *+* | SA-78 | *mecA, ermC* | *+* | SA-144 | *mecA* | *+* |
| SA-44 | *ermA, mecA,* | *+* | SA-79 | *ermA, mecA* | *+* | SA-148 | *ermC, mecA* | *+* |
| SA-45 | *ermA, ermC, mecA* | *+* | SA-80 | *ermC, mecA* | *+* | SA-151 | *mecA* | *+* |
| SA-47 | *mecA* | *+* | SA-81 | *mecA, ermC, msrA* | *+* | SA-153 | *mecA, ermC, msrA* | *+* |
| SA-48 | *ermA, mecA* | *+* | SA-82 | *ermA, ermC, mecA* | *+* |  |  |  |
| SA-50 | *mecA, msrA* | *+* | SA-84 | *ermC, mecA* | *+* |  |  |  |
| SA-51 | *mecA* | *+* | SA-87 | *mecA, msrA* | *+* |  |  |  |
| SA-52 | *ermA, mecA* | *+* | SA-88 | *ermC, mecA* | *+* |  |  |  |
| SA-54 | *ermC, mecA* | *+* | SA-89 | *mecA* | *+* |  |  |  |

*erm*A, erythromycin 23S ribosomal methylase gene A; *erm*C, erythromycin 23S ribosomal methylase gene C; *msr*A, MAC-streptogramin efflux resistance gene; *mec*A, encodes the protein PBP2A (penicillin-binding protein 2A); *nuc,* thermostable nuclease.

**Table S3**. The Fractional Inhibitory concentration (FIC) values of the five pristinamycin (PST)-antibiotic combinations.

| **Isolate code** | **MIC (µg/mL)** | | | **PST + FOX** | | **MIC (µg/mL) LNZ** | | **PST + LNZ** | | **MIC (µg/mL) LEV** | | **PST +LEV** | | **MIC (µg/mL) CN** | | **PST + CN** | | **MIC (µg/mL) DO** | | **PST+ DO** | |
| --- | --- | --- | --- | --- | --- | --- | --- | --- | --- | --- | --- | --- | --- | --- | --- | --- | --- | --- | --- | --- | --- |
|  | **PST** | **FOX** | Σ**FIC index** | |  | | Σ**FIC index** | |  | | Σ**FIC index** | |  | | Σ**FIC index** | |  | | Σ**FIC index** | |  |
| SA-1 | R/8 | R/8 | 2.0(I) | | S/2 | | 0.75(D) | | S/0.5 | | 0.50(Sy) | | S/1 | | 2.0(I) | | R/16 | | 1.0(D) | |  |
| SA-4 | S/1 | R/32 | 0.75(D) | | S/1 | | 0.50(Sy) | | S/1 | | 0.25(Sy) | | S/4 | | 0.50(Sy) | | R/32 | | 0.50(Sy) | |  |
| SA-6 | S/1 | R/16 | 0.75(D) | | S/4 | | 0.31(Sy) | | S/1 | | 0.25(Sy) | | R/16 | | 1.0(D) | | R/32 | | 0.38(Sy) | |  |
| SA-8 | R/4 | R/16 | 1.5(I) | | R/16 | | 2.25(I) | | R/4 | | 1.5(I) | | R/32 | | 1.25(I) | | S/4 | | 0.50(Sy) | |  |
| SA-12 | R/16 | R/64 | 1.06(I) | | S/0.5 | | 0.75(D) | | R/16 | | 1.25(I) | | R/64 | | 0.75(D) | | R/64 | | 0.75(D) | |  |
| SA-17 | S/0.5 | R/8 | 0.75(D) | | S/1 | | 0.75(D) | | S/0.5 | | 0.50(Sy) | | R/32 | | 2.25(I) | | R/32 | | 0.31(Sy) | |  |
| SA-22 | R/8 | R/128 | 2.0(I) | | S/0.5 | | 0.63(D) | | S/0.125 | | 0.25(Sy) | | S/2 | | 0.75(D) | | S/1 | | 0.50(Sy) | |  |
| SA-23 | S/0.5 | R/16 | 0.75(D) | | S/4 | | 0.50(Sy) | | R/4 | | 1.0(D) | | R/16 | | 1.5(I) | | S/2 | | 0.25(Sy) | |  |
| SA-25 | R/8 | R/8 | 1.25(I) | | S/2 | | 0.63(D) | | R/16 | | 1.5(I) | | R/16 | | 2.0(I) | | S/4 | | 0.50(Sy) | |  |
| SA-33 | S/1 | R/32 | 1.5(I) | | S/2 | | 0.38(Sy) | | R/16 | | 0.50(Sy) | | R/32 | | 0.75(D) | | R/16 | | 0.50(Sy) | |  |
| SA-34 | R/32 | R/32 | 1.25(I) | | S/2 | | 1.0(D) | | S/0.125 | | 0.25(Sy) | | S/4 | | 1.25(I) | | S/1 | | 0.25(Sy) | |  |
| SA-36 | S/1 | R/16 | 0.63(D) | | S/1 | | 0.75(D) | | S/0.5 | | 0.50(Sy) | | S/2 | | 0.31(Sy) | | S/1 | | 0.25(Sy) | |  |
| SA-38 | R/16 | R/64 | 1.25(I) | | S/4 | | 2.25(I) | | R/8 | | 1.25(I) | | R/64 | | 0.75(D) | | R/32 | | 0.75(D) | |  |
| SA-39 | S/1 | R/256 | 1.13(I) | | S/0.25 | | 0.75(D) | | R/8 | | 0.50(Sy) | | R/64 | | 1.5(I) | | S/4 | | 0.50(Sy) | |  |
| SA-41 | R/32 | R/32 | 2.5(I) | | S/0.5 | | 0.63(D) | | S/1 | | 0.75(D) | | S/0.5 | | 2.25(I) | | S/1 | | 0.50(Sy) | |  |
| SA-42 | S/1 | R/16 | 2.0(I) | | S/0.25 | | 0.50(Sy) | | S/1 | | 0.38(Sy) | | S/1 | | 0.50(Sy) | | S/2 | | 0.25(Sy) | |  |
| SA-44 | S/0.5 | R/32 | 1.0(D) | | R/16 | | 0.75(D) | | R/8 | | 1.0(D) | | R/64 | | 0.75(D) | | R/32 | | 0.50(Sy) | |  |
| SA-45 | S/0.5 | R/64 | 1.13(I) | | S/4 | | 0.31(Sy) | | S/0.25 | | 0.25(Sy) | | S/2 | | 0.50(Sy) | | S/1 | | 0.25(Sy) | |  |
| SA-47 | S/0.5 | R/8 | 0.5(Sy) | | S/2 | | 0.75(D) | | S/0.25 | | 0.25(Sy) | | S/4 | | 0.38(Sy) | | S/1 | | 0.25(Sy) | |  |
| SA-48 | S/1 | R/32 | 1.06(I) | | S/1 | | 0.75(D) | | S/0.5 | | 0.25(Sy) | | S/4 | | 0.50(Sy) | | S/4 | | 0.38(Sy) | |  |
| SA-50 | R/16 | R/16 | 2.5(I) | | S/0.5 | | 0.63(D) | | R/32 | | 0.31(Sy) | | R/16 | | 1.25(I) | | R/16 | | 0.75(D) | |  |
| SA-51 | S/1 | R/32 | 1.5(I) | | S/0.5 | | 0.63(D) | | S/1 | | 0.50(Sy) | | S/0.5 | | 0.25(Sy) | | S/1 | | 0.50(Sy) | |  |
| SA-52 | S/0.5 | R/256 | 2.5(I) | | R/8 | | 0.63(D) | | R/8 | | 0.25(Sy) | | R/32 | | 1.0(D) | | R/32 | | 0.31(Sy) | |  |
| SA-54 | R/16 | R/8 | 1.13(I) | | S/4 | | 0.63(D) | | S/0.5 | | 0.50(Sy) | | S/0.125 | | 2.25(I) | | S/4 | | 0.25(Sy) | |  |
| SA-55 | S/1 | R/16 | 1.5(I) | | S/1 | | 0.50(Sy) | | S/1 | | 0.25(Sy) | | S/1 | | 0.50(Sy) | | S/1 | | 0.25(Sy) | |  |
| SA-57 | R/8 | R/32 | 1.25(I) | | S/1 | | 0.63(D) | | R/8 | | 1.5(I) | | R/64 | | 1.5(I) | | R/16 | | 0.75(D) | |  |
| SA-58 | R/16 | R/16 | 1.5(I) | | S/0.25 | | 2.0(I) | | S/0.5 | | 0.50(Sy) | | S/1 | | 0.75(D) | | S/2 | | 0.25(Sy) | |  |
| SA-59 | S/1 | R/128 | 0.75(D) | | S/2 | | 0.63(D) | | R/8 | | 0.50(Sy) | | R/64 | | 2.0(I) | | R/32 | | 0.50(Sy) | |  |
| SA-61 | S/0.5 | R/64 | 1.13(I) | | R/16 | | 1.0(D) | | R/16 | | 0.25(Sy) | | R/64 | | 1.0(D) | | R/16 | | 0.50(Sy) | |  |
| SA-62 | R/16 | R/8 | 1.25(I) | | S0.5 | | 0.63(D) | | S/1 | | 0.36(Sy) | | S/1 | | 2.0(I) | | R/16 | | 0.75(D) | |  |
| SA-63 | R/4 | R/128 | 2.5(I) | | S/4 | | 0.75(D) | | R/64 | | 1.25(I) | | R/128 | | 1.5(I) | | S/4 | | 0.25(Sy) | |  |
| SA-64 | S/1 | R/8 | 0.50(Sy) | | S/1 | | 1.0(D) | | R/16 | | 0.50(Sy) | | R/64 | | 1.0(D) | | S/1 | | 0.25(Sy) | |  |
| SA-66 | S/0.5 | R/128 | 1.13(I) | | S0.5 | | 0.50(Sy) | | R/8 | | 0.25(Sy) | | R/32 | | 0.75(D) | | S/2 | | 0.50(Sy) | |  |
| SA-67 | R/16 | R/64 | 2.0(I) | | S/2 | | 1.0(D) | | S/0.5 | | 0.50(Sy) | | S/4 | | 1.25(I) | | S/1 | | 0.25(Sy) | |  |
| SA-69 | S/1 | R/64 | 1.5(I) | | S/1 | | 0.75(D) | | R/16 | | 0.25(Sy) | | R/16 | | 1.0(D) | | S/1 | | 0.25(Sy) | |  |
| SA-70 | R/4 | R/8 | 1.13(I) | | S/4 | | 0.63(D) | | S/1 | | 0.25(Sy) | | R/256 | | 1.0(D) | | S/4 | | 0.38(Sy) | |  |
| SA-73 | S/1 | R/16 | 2.0(I) | | S/2 | | 0.75(D) | | R/16 | | 1.0(D) | | R/64 | | 1.25(I) | | S/2 | | 0.38(Sy) | |  |
| SA-75 | R/4 | R/64 | 1.25(I) | | R/16 | | 1.06(I) | | R/64 | | 0.25(Sy) | | R/64 | | 0.75(D) | | S/1 | | 0.25(Sy) | |  |
| SA-77 | R/16 | R/8 | 1.5(I) | | S/4 | | 0.75(D) | | S/0.5 | | 0.75(D) | | R/16 | | 3.0(I) | | S/4 | | 0.50(Sy) | |  |
| SA-78 | S/1 | R/128 | 1.0(D) | | S/2 | | 1.0(D) | | S/1 | | 0.25(Sy) | | R/16 | | 0.25(Sy) | | S/1 | | 0.25(Sy) | |  |
| SA-79 | S/0.5 | R/8 | 0.63(D) | | R/16 | | 2.25(I) | | R/16 | | 0.25(Sy) | | R/64 | | 0.63(D) | | S/4 | | 0.31(Sy) | |  |
| SA-80 | S/0.5 | R/64 | 0.63(D) | | S/1 | | 0.50(Sy) | | R/64 | | 0.125(Sy) | | R/64 | | 0.75(D) | | S/1 | | 0.25(Sy) | |  |
| SA-81 | R/4 | R/128 | 3.0(I) | | S/4 | | 1.0(D) | | R/32 | | 1.25(I) | | R/256 | | 1.13(I) | | S/1 | | 0.25(Sy) | |  |
| SA-82 | R/8 | R/8 | 1.13(I) | | S/0.5 | | 0.63(D) | | S/1 | | 0.38(Sy) | | R/16 | | 1.25(I) | | R/16 | | 0.75(D) | |  |
| SA-84 | S/0.5 | R/64 | 1.0(D) | | S/2 | | 0.50(Sy) | | R/8 | | 0.63(D) | | R/256 | | 0.75(D) | | S/2 | | 0.36(Sy) | |  |
| SA-87 | R/4 | R/64 | 2.0(I) | | S/1 | | 0.63(D) | | S/0.25 | | 0.25(Sy) | | R/512 | | 2.5(I) | | S/1 | | 0.25(Sy) | |  |
| SA-88 | R/8 | R/8 | 1.5(I) | | S/4 | | 0.63(D) | | S/0.125 | | 0.25(Sy) | | R/32 | | 2.5(I) | | S/1 | | 0.38(Sy) | |  |
| SA-89 | S/0.5 | R/128 | 2.5(I) | | S/0.5 | | 1.0(D) | | R/128 | | 0.75(D) | | R/16 | | 1.0(D) | | S/4 | | 0.25(Sy) | |  |
| SA-95 | R/16 | R/16 | 1.5(I) | | S/2 | | 1.06(I) | | S/0.5 | | 0.25(Sy) | | R/32 | | 1.0(D) | | S/4 | | 0.31(Sy) | |  |
| SA-99 | R/4 | R/8 | 2.5(I) | | S/2 | | 1.0(D) | | S/1 | | 0.75(D) | | R/64 | | 1.13(I) | | S/4 | | 0.25(Sy) | |  |
| SA-101 | R/8 | R/64 | 1.06(I) | | S/4 | | 2.25(I) | | R/16 | | 1.5(I) | | R/32 | | 2.25(I) | | R/16 | | 0.75(D) | |  |
| SA-107 | R/16 | R/8 | 2.25(I) | | S/1 | | 0.63(D) | | S/0.125 | | 0.31(Sy) | | R/16 | | 1.5(I) | | S/1 | | 0.25(Sy) | |  |
| SA-114 | S/0.5 | R/8 | 0.5(Sy) | | S/4 | | 1.0(D) | | S/1 | | 0.25(Sy) | | R/128 | | 2.5(I) | | S/1 | | 0.25(Sy) | |  |
| SA-115 | S/1 | R/32 | 1.0(D) | | S/2 | | 0.50(Sy) | | S/0.125 | | 0.25(Sy) | | R/16 | | 1.0(D) | | S/0.5 | | 0.25(Sy) | |  |
| SA-118 | S/0.25 | R/64 | 0.63(D) | | R/8 | | 1.0(D) | | S/0.5 | | 0.38(Sy) | | R/32 | | 2.5(I) | | R/16 | | 0.75(D) | |  |
| SA-120 | S/0.5 | R/8 | 0.36(Sy) | | S/0.5 | | 0.25(Sy) | | S/1 | | 0.38(Sy) | | R/256 | | 0.75(D) | | S/4 | | 0.50(Sy) | |  |
| SA-122 | R/4 | R/8 | 1.5(I) | | S/2 | | 1.0(D) | | S/0.125 | | 0.25(Sy) | | R/64 | | 2.0(I) | | S/2 | | 0.50(Sy) | |  |
| SA-127 | S/0.5 | R/8 | 1.5(I) | | S/2 | | 0.50(Sy) | | S/0.5 | | 0.50(Sy) | | R/16 | | 1.5(I) | | R/32 | | 0.25(Sy) | |  |
| SA-129 | S/1 | R/128 | 1.25(I) | | S/4 | | 0.63(D) | | R/8 | | 0.75(D) | | R/64 | | 2.5(I) | | S/4 | | 0.31(Sy) | |  |
| SA-132 | R/4 | R/64 | 1.25(I) | | S/1 | | 0.75(D) | | R/16 | | 1.5(I) | | R/32 | | 2.0(I) | | R/32 | | 0.75(D) | |  |
| SA-134 | S/1 | R/8 | 0.63(D) | | S/2 | | 0.31(Sy) | | S/1 | | 0.31(Sy) | | R/16 | | 0.75(D) | | R/16 | | 0.63(D) | |  |
| SA-139 | R/8 | R/64 | 2.0(I) | | S/2 | | 0.75(D) | | S/1 | | 0.50(Sy) | | R/32 | | 1.5(I) | | R/16 | | 1.0(D) | |  |
| SA-142 | S/0.5 | R/16 | 1.25(I) | | S/4 | | 0.38(Sy) | | R/8 | | 0.25(Sy) | | R/16 | | 0.25(Sy) | | S/2 | | 0.50(Sy) | |  |
| SA-144 | S/1 | R/8 | 0.38(Sy) | | S/2 | | 1.0(D) | | S/0.5 | | 0.38(Sy) | | R/64 | | 2.5(I) | | R/32 | | 0.25(Sy) | |  |
| SA-148 | R/8 | R/8 | 1.5(I) | | S/1 | | 0.63(D) | | R/16 | | 1.25(I) | | R/16 | | 2.0(I) | | S/4 | | 0.38(Sy) | |  |
| SA-151 | S/0.5 | R/16 | 1.06(I) | | R/8 | | 0.63(D) | | S/1 | | 0.25(Sy) | | R/16 | | 1.5(I) | | R/16 | | 0.31(Sy) | |  |
| SA-153 | R/16 | R/16 | 1.5(I) | | R/8 | | 0.75(D) | | R/16 | | 1.5(I) | | R/64 | | 2.0(I) | | S/2 | | 0.25(Sy) | |  |
| Synergy |  |  | 5 (7.64%) | |  | | 15 (22.8%) | |  | | 47 (70.14%) | |  | | 10 (14.9%) | |  | | 55 (82.0%) | |  |
| Additive |  |  | 14 (20.8%) | |  | | 45 (67%) | |  | | 9 (13.4%) | |  | | 22 (32.8%) | |  | | 12 (17.9%) | |  |
| Indifferent |  |  | 48 (71.6%) | |  | | 7 (10.4%) | |  | | 11 (16.4%) | |  | | 35 (52.2%) | |  | | 0 (0.0%) | |  |

PST, pristinamycin; FOX, cefoxitin; LNZ, linezolid; LEV, levofloxacin, CN, gentamicin; DO, doxycycline; (Sy) Synergism ≤0.5; (D), Additive >0.5 ≥1; (I), Indifference >1 and ≤4.0; FIC, fractional inhibitory concentration. S, sensitive; R, resistance.
